# The cresting wave: larval settlement and ocean temperatures predict change in the American lobster harvest

**DOI:** 10.1002/eap.2006

**Published:** 2019-10-21

**Authors:** Noah G. Oppenheim, Richard A. Wahle, Damian C. Brady, Andrew G. Goode, Andrew J. Pershing

**Affiliations:** ^1^ University of Maine School of Marine Sciences Darling Marine Center Walpole Maine 04573 USA; ^2^ Institute for Fisheries Resources 991 Marine Drive San Francisco California 94129 USA; ^3^ Gulf of Maine Research Institute Commercial Street Portland Maine 04101 USA

**Keywords:** climate adaptation, crustacean, environmental gradients, fisheries, forecasting, Gulf of Maine, *Homarus americanus*, lobster, recruitment processes, regional downscaling

## Abstract

Adding to the challenge of predicting fishery recruitment in a changing environment is downscaling predictions to capture locally divergent trends over a species’ range. In recent decades, the American lobster (*Homarus americanus*) fishery has shifted poleward along the northwest Atlantic coast, one of the most rapidly warming regions of the world's oceans. Building on evidence that early post‐settlement life stages predict future fishery recruitment, we describe enhancements to a forecasting model that predict landings using an annual larval settlement index from 62 fixed sites among 10 study areas from Rhode Island, USA to New Brunswick, Canada. The model is novel because it incorporates local bottom temperature and disease prevalence to scale spatial and temporal changes in growth and mortality. For nine of these areas, adding environmental predictors significantly improved model performance, capturing a landings surge in the eastern Gulf of Maine, and collapse in southern New England. On the strength of these analyses, we project landings within the next decade to decline to near historical levels in the Gulf of Maine and no recovery in the south. This approach is timely as downscaled ocean temperature projections enable decision makers to assess their options under future climate scenarios at finer spatial scales.

## Introduction

Rapid climate change in coastal ecosystems increases the urgency to develop forecasting tools enabling fishing communities and resource managers to anticipate and adapt to distributional shifts in the abundance of target species (FAO 2016, Payne 2017). In many fisheries, insufficient data are available on critical life stages or the environment, and over a large enough spatial scale, to give advance warning of geographic shifts in recruitment. A standard practice in fisheries science is to project future recruitment from assumed spawner–recruit relationships that are notoriously variable (Myers 1998, Wahle 2003). Our project is predicated on the assumption that, for relatively long‐lived benthic species with pelagic larvae, monitoring the abundance of a post‐settlement stage that has already passed through the larval gauntlet can be a valuable predictor of the abundance of fishery recruits several years in the future (Caputi et al. 1995, Pineda 2000). A further benefit is that tracking preharvest life stages to the time they recruit to the fishery sidesteps the complicating effects of fishing mortality. Therefore, early life stage monitoring across significant geographic environmental gradients, or periods of demographic change, can offer insight into the role of the environment in determining year‐class strength and fishery recruitment. The challenge with long‐lived species is to develop and validate mechanistic predictive models that incorporate key drivers influencing the fate of cohorts from within weeks of hatching to recruitment to the fishery years later.

The American lobster (*Homarus americanus*) currently supports the most valuable single‐species fishery in the United States and Canada, with a combined landed value exceeding $US1 billion in 2016. Its geographic range from Newfoundland Canada to the mid‐Atlantic shelf of the USA spans one of the steepest latitudinal gradients in sea surface temperature in the world (Longhurst 2006). This region has been warming faster than most of the world's ocean (Pershing 2015), resulting in poleward shifts in the geographic range of numerous commercially important marine species, including lobster (Nye et al. 2009, Pinsky et al. 2013). Just as lobster populations have collapsed and receded from historically productive grounds in southern New England, largely due to the adverse effects of warming (Pearce and Balcom 2005, Wahle et al. 2009, 2015), populations further north in the Gulf of Maine and Bay of Fundy have undergone unprecedented expansion resulting in a three‐ to fourfold increase in total lobster production since the 1990s that has elevated the fishery to its premiere status (NOAA 2017). In Maine, where lobster comprise approximately three‐quarters of total fishery revenue, harvesters have few alternative fisheries should there be a downturn (Steneck 2011). These shifting economic dependencies heighten the need to understand and predict future change.

The present study is the third in a series of recently emerging forecasting products applied to the American lobster that range from short‐term, seasonal‐scale (Mills et al. 2017) to long‐term, decadal‐scale forecasts (Le Bris et al. 2018). Under likely global change scenarios, the decadal scale, multigenerational, forecasts suggest the wave of high lobster abundance New England is now enjoying will pass by as favorable environmental conditions shift northward in the coming decades. The American lobster fishery is managed primarily through size limits, effort controls through trap limits, season length, and prohibitions on the take of egg bearing females (ASMFC 2015). Application of environmental indicators in management has been limited to date, but nonetheless recognized as important to a fishery more adaptive to environmental change. The decadal‐scale forecasting model described by Le Bris et al. (2018), for example, suggests relatively small adjustments to these conservation measures could help the fishery capitalize on favorable conditions and forestall collapse under environmental stress.

Long‐term forecasting, however, is an inherently uncertain enterprise. Missing are near‐term, finer spatial scale, projections that could help decision makers corroborate and localize long‐term projections. The present study describes a modeling framework that brings the fate of individual cohorts into focus at a time scale of 5–10 yr at more local scale that may be more meaningful to shorter term interests of fishery managers and the fishing industry. Building on an approach developed by Wahle et al. (2004, 2009) to predict recruitment to the lobster fishery using a larval settlement index, we project recruitment to the fishery based on an empirical understanding of environmental effects on growth and mortality that influence the fractional contribution of multiple cohorts to total fishery recruitment in a given year.

Encouraged by initial success in settlement‐based forecasting at one of our study areas (Wahle et al. 2009), the present study encompasses ten study areas of similar size but contrasting thermal regimes from southern New England to the Bay of Fundy. We validate our settlement‐based predictions of fishery recruitment against observed lobster landings for each reporting area. On the strength of these relationships we project landings as much as 7 yr into the future. Our forecast projects future declines consistent with those projected in Le Bris's longer‐term forecast, having important implications especially for the eastern Gulf of Maine, the region that elevated the fishery to its current premier status over the past decade. It also has the advantage identifying sub‐stock scale differences recruitment not available with the Le Bris model. We believe this approach may be more widely applied to any long‐lived species for which data time series of early life stages and environmental drivers of growth and mortality are available, enabling resource managers to anticipate localized changes in fishery productivity.

## Methods

### Data sources

**Figure 1:**
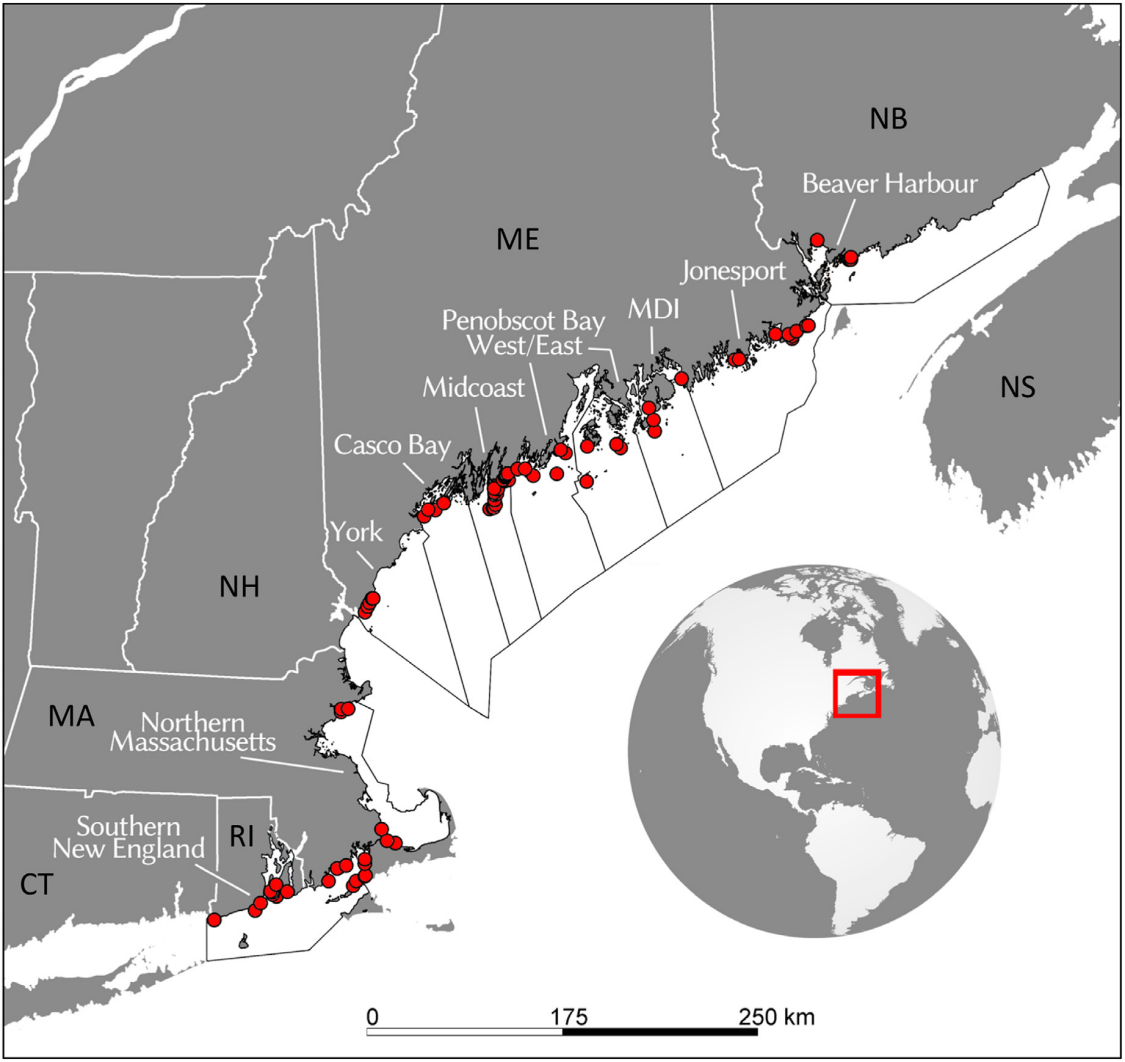
American Lobster Settlement Index sites (red) within study areas (labeled in white); corresponding statistical reporting areas outlined in black. CT, Connecticut, USA; RI, Rhode Island, USA; MA, Massachusetts, USA; NH, New Hampshire, USA; ME, Maine, USE; NB, New Brunswick, Canada; NS, Nova Scotia, Canada.

**Figure 2:**
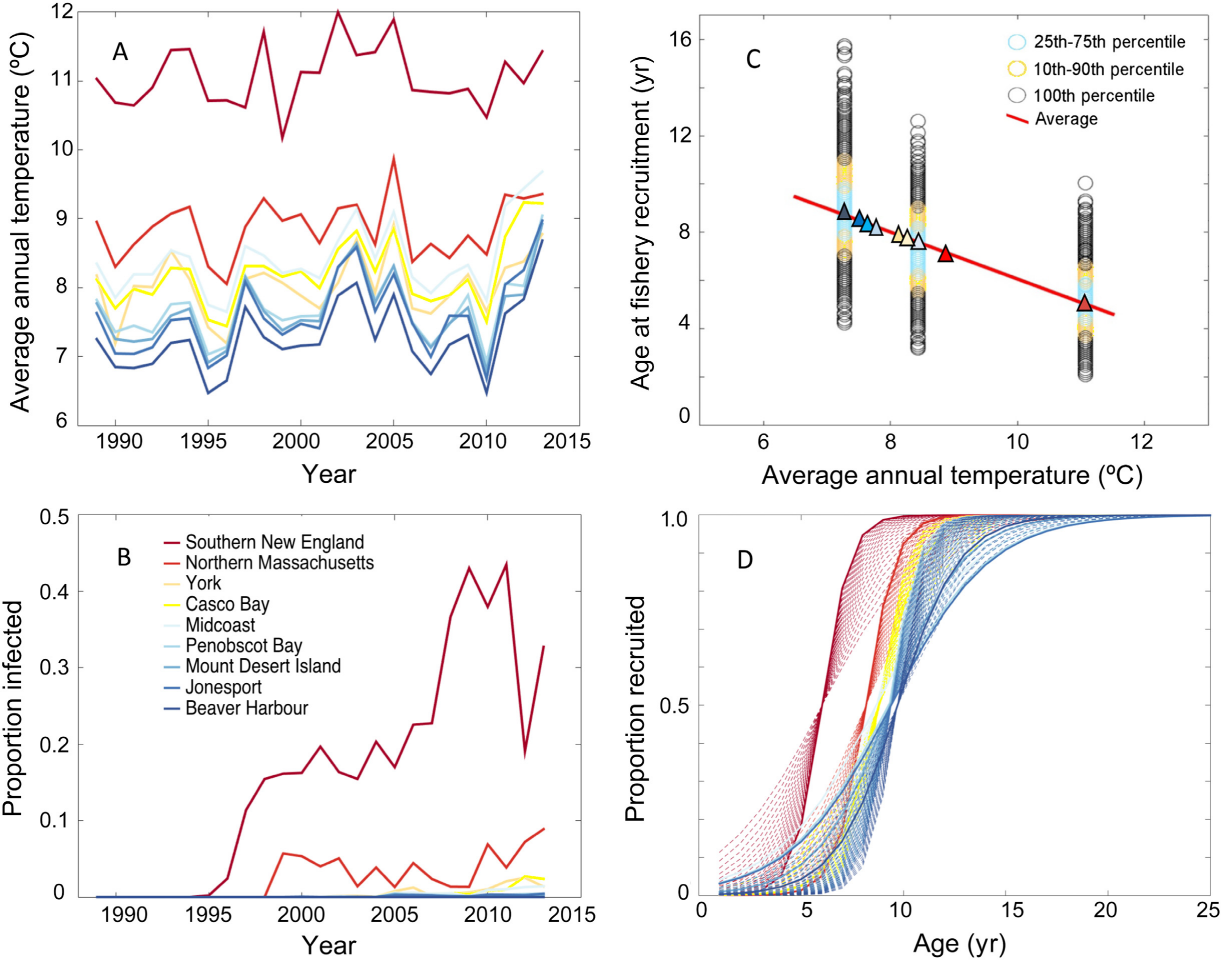
Environmental variables used in model development. (A) Annual bottom temperature time series from the NECOFS FVCOM‐GOM3 hindcast model in 10‐km^2^ grids adjacent to ALSI study areas. East and west Penobscot Bay study areas share a single temperature time series. (B) Shell disease prevalence in lobsters up to 83 mm in commercial catch by reporting area. (C) Estimated age at 50% fishery recruitment vs. mean bottom temperature. Monte Carlo simulated distributions of age at fishery recruitment for three thermally contrasting study areas with empirical growth data (Beaver Harbour, midcoast Maine, southern New England) regressed against mean annual bottom temperatures (°C) for those areas. Resulting equation (Appendix S1: Eq. S3) used to estimate age‐at‐fishery‐recruitment for areas without empirical growth data. Triangles denote model‐estimated age at fishery recruitment for each study area. (D) Logistic age‐at‐fishery‐recruitment curves for study areas where predicted recruitment trends significantly correlated with reported landings. Best fitting logistic models denoted by solid line; dashed lines denote range of statistically significant curves. Legend applies to all. Model assessment in Table 1.

Detailed explanation of data sources used in this study is available in Appendix S1. Briefly, the American Lobster Settlement Index (ALSI) forms the basis of annual cohort (year‐class) strength from which fishery recruitment predictions are made. This annual indicator of the abundance of newly settled young‐of‐year (YoY) lobsters provides a relative measure of year‐class strength for this species. We used survey data from 62 fixed sampling sites spread among 10 study areas from southern New England to the Bay of Fundy (Fig. 1). Densities were averaged among sites to give a study area mean YoY density ± SE (Appendix S1: Fig. S1). We used landings trends for each study area as a proxy for fishery recruitment to validate ALSI‐based fishery recruitment predictions (Appendix S1: Fig. S2), and justify their use as such based on evidence that the annual harvest largely comprises new recruits to the fishery (ASMFC 2015), and a region‐wide analysis indicating lobster abundance trends lead, rather than follow, trends in fishing effort (Boudreau et al. 2015). Temperature strongly influences lobster growth (Wahle and Fogarty 2006). We used the Northeast Coastal Ocean Forecast System (NECOFS) Finite Volume Community Ocean Model for the Gulf of Maine (FVCOM‐GOM) 30‐yr hindcast model to generate annual average bottom temperature time series for each ALSI study area (Fig. 2a). Li et al. (2017) describes a thorough skill assessment of the FVCOM‐modeled bottom temperatures in coastal waters through a comparison to in situ measures of bottom temperatures. For lack of empirical estimates of natural mortality, we used a baseline annual proportional mortality of 0.139 yr^−1^ equivalent to the instantaneous rate of 0.15 yr^−1^ (ASMFC 2015). Shell disease represents an added source of mortality that became prevalent in southern New England during the late 1990s (Wahle et al. 2009). Survey estimates of disease prevalence (Fig. 2b) were incorporated in the model by addition to the baseline mortality.

### Predictive model

All modeling and statistical analysis were conducted in (MatLab v.9.0, Natick, MA, USA). Our modeling approach involved two steps that are also detailed in Appendix S1. First, we developed a process model that produced an annual fishery recruitment index by summing the fractional contributions of several settlement year‐classes as they enter the fishery some 5–10 yr after settlement. This involved the novel approach of scaling the natural mortality term (Fig. 2b) and logistic growth function (Fig. 2c, d) to area‐specific conditions. The process model also employed a Monte Carlo method to estimate uncertainty in the annual recruitment index using observed variability in settlement index and growth rates for each study area.

Next, we employed a validation step that statistically compared the predicted recruitment time series to observed landings. If this step resulted in a significant linear relationship, the regression parameters were used to project landings using the fishery recruitment index. If a study area failed the validation step, we discontinued analysis of that area. Finally, to further assess the skill of the model at predicting landings in the out years, we compared the projections to observed landings in 2016 and 2017. The key enhancements to earlier versions of the model were to incorporate temperature‐dependent growth, to adjust the base natural mortality rate to account for shell disease, to convert the estimated recruitment index to an estimate of landings, and to estimate uncertainty around the prediction.

To illustrate the benefits of applying the logistic growth model and a base mortality term, we ran the fishery recruitment model for each study area under six modeling scenarios. For three “sentinel” study areas with the longest settlement time series (Beaver Harbour, New Brunswick, Canada; midcoast Maine, USA; and southern New England) we compared model performance using (1) a simple rolling average of the settlement index lagged by mean age at fishery recruitment and no mortality, and (2) a globally fixed single logistic growth function determined for midcoast Maine and the baseline mortality term (0.15 yr^−1^). Then, for all study areas, we developed locally tuned area‐specific models with (3) the logistic growth functions scaled to a NECOFS‐modeled fixed average temperature for the study area, (4) the growth function varying at each time step according to annually varying bottom temperature for the study area, (5) bottom temperature fixed + disease prevalence reported for the study area, and (6) bottom temperature variable + disease prevalence.

For each of the six progressively more complex scenarios, we validated model hindcasts by regressing the predicted recruitment index against observed landings from corresponding statistical reporting areas or management zones through 2017. We used linear regressions with gamma probability distributions to provide correlation coefficients *r* and significance levels. Gamma distributions were used because forecast indices were positive, heteroskedastic, and right skewed. We selected the most parsimonious model by sequential consideration of low Akaike information criterion (AIC; Burnham and Anderson 2003) values, low *P* values, and high *r* values. The AIC explicitly addresses model parsimony by penalizing models with the greater number of parameters. Landings forecasts for years beyond 2017 were generated using the regression coefficients from the best fitting hindcasts to translate the recruitment index into projected landings**.** Finally, we conducted an out‐of‐sample skill assessment of the model in each study area. To do this we created models that iteratively omitted from one to four of the most recent settlement years to evaluate how well the model would perform had those years not been included in the model.

## Results

### Hindcasts and model validation

We found statistically significant positive linear relationships between the YoY recruitment index hindcasts and observed landings time series for 9 of the 10 study areas (Table 1). Models that scaled the logistic growth function and the mortality term to the study area's temperature and disease prevalence, respectively, consistently improved model performance. For the three “sentinel” study areas with the longest time series, the higher *r* values and lower *P* and AIC values indicate the recruitment model with the logistic function and baseline mortality consistently outperformed the relatively simple rolling average approach. Applying area‐specified temperatures to tune the logistic growth model, either with the fixed time series average or varying by year, further improved model fit for the nine areas. While the most parsimonious model for each study area, based on high *r* and low *P* and AIC scores, is indicated in boldface in Table 1, for all practical purposes there was little difference in performance between the fixed temperature and annually varying temperature versions of the model. In two cases (Jonesport, Maine and northern Massachusetts), where AIC scores for fixed and variable temperature models were equal, model selection was based on the higher and more significant *r* values. In one case, York, Maine, we found no statistically significant relationship between YoY recruitment and landings, and this study area was excluded from further analysis. Finally, adding shell disease to the mortality term dramatically improved model fit for southern New England, where the disease was most prevalent, and for the other study areas, including disease added no additional predictive power. Model fit was relatively insensitive to varying the logistic function slope parameter (*b*) because a broad range of tested values gave statistically significant correlations with landings through 2017 (Appendix S1: Table S3).

**Table 1:** Recruitment index‐to‐landings correlation results

							Area‐specific											
Study area	Rolling average settlement			Globally fixed temp			Fixed temp			Variable temp			Fixed temp + disease			Variable temp + disease		
	*r*	*P*	AIC	*r*	*P*	AIC	*r*	*P*	AIC	*r*	*P*	AIC	*r*	*P*	AIC	*r*	*P*	AIC
Beaver Harbour, NB	0.89	<0.0001	516	0.92	<0.0001	482	**0.97**	**<0.0001**	**469**	0.97	<0.0001	472	0.97	<0.0001	470	0.97	<0.0001	473
Jonesport, ME							0.90	0.0017	256	**0.92**	**0.0010**	**256**	0.90	0.0013	257	0.91	0.0012	258
MDI, ME							**0.97**	**<0.0001**	**402**	0.97	<0.0001	404	0.97	<0.0001	405	0.98	<0.0001	405
Penobscot Bay E, ME							**0.95**	**<0.0001**	**291**	0.95	<0.0001	293	0.95	<0.0001	294	0.94	0.0001	296
Penobscot Bay W, ME							**0.80**	**0.0087**	**286**	0.79	0.0103	288	0.82	0.0052	286	0.80	0.0084	290
Midcoast, ME	0.39	0.0980	630	0.75	0.0002	561	**0.75**	**0.0002**	**561**	0.75	0.0002	564	0.74	0.0003	564	0.75	0.0003	566
Casco Bay, ME							**0.72**	**0.0217**	**278**	0.69	0.0279	280	0.69	0.0338	280	0.68	0.0308	283
York, ME							−0.45	0.2169	262	−0.28	0.4733	265	−0.50	0.1582	263	−0.39	0.3058	266
N MA							0.70	0.0027	421	**0.75**	**0.0008**	**421**	0.65	0.0060	425	0.72	0.0016	424
S New England	0.42	0.0670	632	0.45	0.1460	626	0.48	0.0989	625	0.52	0.0645	625	0.69	0.0102	612	**0.71**	**0.0076**	**611**

*Notes*

**Figure 3:**
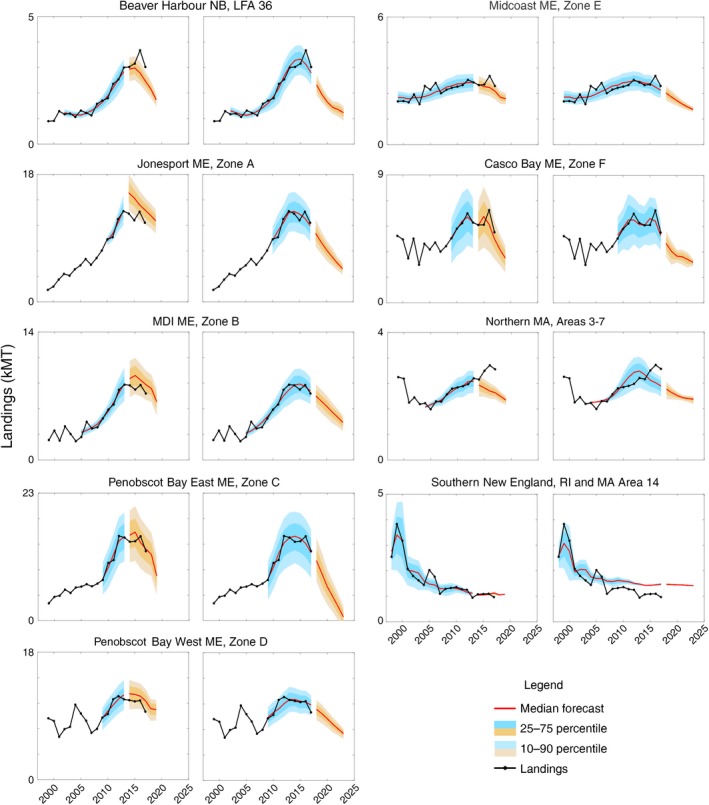
Predictive model hindcasts and forecasts for the nine study areas with significant recruitment index‐to‐landings relationships using the best‐fitting models listed in boldface type in Table 1. Compared for each study area are the full model using all years of data available through 2017 (right panel) to our out‐of‐sample skill assessment excluding the last 4 yr of data to 2013. Observed (black line) and predicted (red line) lobster landings, hindcasts (blue), and forecasts from 2013 forward (orange) with 25–75% (dark shaded) and 10–90% (light shaded) quantiles. Skill assessment for intervening years shown in Table 2. See Fig. 1 for state and province names.

Correlation coefficient (*r*), significance levels (*P*) and Akaike information criterion (AIC) indices for linear regressions of fishery recruitment indexes against landings using six increasingly complex model versions for the ten study areas with young‐of‐the‐year recruitment data through 2017. The most parsimonious model for each study area is indicated in boldface type and depicted in Fig. 3. The globally fixed model applied the Midcoast ME parameters to the study areas at the geographic extremes (southern New England and Beaver Harbour, NB). York was excluded from subsequent analysis because of nonsignificant correlation. Abbreviations are N, northern; S, southern; E, east; W, west; NB, New Brunswick, Canada; ME, Maine, USA; MA, Massachusetts, USA; temp, temperature.

For the nine areas with significant positive statistical fits, observed landings typically fell well within the 10–90th percentile of landings hindcast distributions, although the model underpredicted landings in northern Massachusetts over the last two years, and it slightly overpredicted landings for southern New England over the last decade (Fig. 3a, Table 2). Nonetheless, the best fitting models captured the dramatic ~400% increase in landings in the eastern Gulf of Maine from New Brunswick to eastern Penobscot Bay, a more modest ~50% increase in the western Gulf of Maine from western Penobscot Bay to Massachusetts, and the ~75% decline in southern New England. The margin of uncertainty was typically proportional to the magnitude of predicted landings and was driven largely by among‐site variability in YoY density within study areas (Fig. 3).

**Table 2:** Out‐of‐sample model skill assessment

Study area	Observed 2017 landings (Metric Tons ×1000)	Year of model projection									
		2017		2016		2015		2014		2013	
		Predicted 2017 landings	Diff.	Predicted 2017 landings	Diff.	Predicted 2017 landings	Diff.	Predicted 2017 landings	Diff.	Predicted 2017 landings	Diff.
Beaver Harbour, NB	3.02	2.79	−0.23	2.72	−0.29	2.58	−0.44	2.48	−0.53	2.46	−0.56
Jonesport, ME	11.16	10.75	−0.42	10.61	−0.55	10.43	−0.74	11.13	−0.04	12.99	1.82
MDI, ME	7.25	7.53	0.28	7.54	0.29	7.27	0.02	7.63	0.38	8.23	0.98
Penobscot Bay E, ME	12.56	12.27	−0.28	12.77	0.21	11.73	−0.82	11.47	−1.08	12.93	0.37
Penobscot Bay W, ME	9.61	10.65	1.03	10.88	1.26	10.91	1.29	10.68	1.06	11.14	1.53
Midcoast ME	2.75	2.56	−0.19	2.56	−0.19	2.50	−0.25	2.50	−0.25	2.52	−0.23
Casco Bay, ME	4.97	4.75	−0.22	4.59	−0.37	4.43	−0.53	2.64	−2.33	4.49	−0.48
N MA	2.84	2.31	−0.53	2.24	−0.60	2.18	−0.66	2.09	−0.75	2.11	−0.73
S New England	0.96	1.47	0.51	1.34	0.38	1.12	0.16	1.13	0.17	1.13	0.17
Mean	6.12	6.12	−0.01	6.14	0.01	5.91	−0.22	5.75	−0.37	6.44	0.32

*Notes*

Comparison of observed 2017 landings to model‐predicted median landings for each study area (excluding York), starting with the full model that includes all years through 2017, to those with progressively more years excluded through 2013 (4 yr excluded). Output for 2017 and 2013 model versions depicted in Fig. 3. Diff., difference. See Table 1 for other abbreviations.

In short, in the majority of cases a modeling approach accounting for temperature‐induced changes in growth rate and a proxy for changing natural mortality successfully linked time trends in settlement to subsequent landings. Although including local differences in temperature clearly improved model prediction, incorporating interannual variability had only a marginal effect and influenced model selection in one case (northern Massachusetts).

### Forecasts and out‐of‐sample model skill assessment

While our settlement‐based forecasts accurately predicted the surge in landings over much of the Gulf of Maine through 2016, as well as the collapse in southern New England, recent declines in settlement at Gulf of Maine study areas portend a downturn in future landings, and continued low settlement in southern New England gives little sign of recovery in the near future. Our out‐of‐sample skill assessment helps test the performance of the model in forecasting future change. Excluding the last four years of data in our out‐of‐sample skill assessment did not dramatically affect model performance for most study areas, except for northern Massachusetts, where the model underpredicted landings in the last 3 yr (Fig. 3). For example, using the model with settlement data up to 2013 we underpredicted landings for Beaver Harbour, New Brunswick, and overpredicted landings for Jonesport, Maine, but including the more recent settlement data has modified that prediction to align with observed landings. On the other hand, we cannot fully account for the continued rise in northern Massachusetts landings beyond 2015 on the basis of ALSI and environmental predictors alone. Curiously, for southern New England, the 2013 model accurately predicted the landings trajectory through 2017, but the full model overpredicted it for the last decade, suggesting changes in landings were unrelated to the most recent trends in previous YoY recruitment. Taken together, on average, with as many as four years excluded, the predicted landings from the best fitting model fell within 5% of observed landings (Table 2).

## Discussion

This study describes an enhanced model developed on the premise that year‐class strength measured at larval settlement, and key environmental indicators influencing demographic rates, can provide useful predictions of subsequent fishery recruitment several years later. To tune the model locally, we used temperature variability and shell disease prevalence to parameterize growth and mortality rates, respectively. Of 10 study areas spanning New England's steep latitudinal temperature gradient, our hindcast validations produced model output for nine areas that significantly correlated with observed landings. We suspect the absence of a significant recruitment index‐to‐landings relationship for York, Maine, is related to the short length of the settlement time series and the minimal contrast in the landings data (Appendix S1: Fig. S2). Furthermore, for eight of the nine areas, observed landings for 2016 and 2017 fell well within the 90% confidence limits of our out‐of‐sample forecasts. Moreover, our out‐of‐sample skill assessment demonstrated good model performance, even with as many as 4 yr excluded in most cases, with the noteworthy exception of our northern Massachusetts study area for which even the full model, including all years, underpredicted the last 2–3 yr of landings. In that case, we suspect either a subsidy of lobsters from the northeast or a change in harvesting area may obscure locally sourced recruitment. Conversely, in the case of southern New England, where the full model overpredicted recent lobster landings, it is possible a shift in fishing effort away from lobster and to alternative fisheries may come into play as the local lobster fishery becomes less economically viable for harvesters.

One benefit of using a settlement index to predict fishery recruitment is that it side‐steps untested and tenuous spawner‐recruit relationships. A similar postlarval settlement index forecasting approach has long been used in the Western Australia rock lobster (*Panulirus cygnus*) fishery, although, in that case, time lags are shorter and thermal gradients less steep (Caputi et al. 1995). Not surprisingly, spawner abundance alone has historically not been a reliable predictor of recruitment variability in most crustaceans (Wahle 2003) and fishes (Schindler and Hilborn 2015), but models that incorporate environmental variability in addition to, or as proxies for, spawner abundance have had more success (Caputi et al. 2014). A longstanding concern with these models, however, is that the relationships between environmental variability and recruitment can be transient (Myers 1998, Schindler and Hilborn 2015). For example, the efficacy of using temperature as a predictor of Pacific sardine recruitment has long been debated (McClatchie et al. 2010). In some cases, strong environmental trends or gradients are needed to provide sufficient contrast to quantify environment–recruitment relationships. For example, Pershing (2015) showed that a temperature‐dependent recruitment model for the Atlantic cod developed during a period of moderate temperature variability (Fogarty et al. 2007) was able to account for a decline in recruitment under a strong warming trend. Further, Myers (1998) hypothesized that fish populations are most responsive to temperature at their range extremes. Our results suggest temperature influences recruitment dynamics of the American lobster over much of its range.

Predictive models such as ours require long time series on early and late life stages and associated environmental data over considerable portions of the species’ range. This approach may have broader application to other fishes and invertebrates with similar life history attributes that vary across environmental gradients in space or time. Similar data are available for a number of fisheries and their environments globally (see FishBase; https://www.fishbase.in/home.htm).^1^ Furthermore, downscaled projections of sea temperature in coastal areas are increasingly available, enabling finer scale projections (Rheuban et al. 2017). The challenge will be to find the set environmental factors representing key drivers of population change into the future.

A bias in the ALSI index could arise if the areal coverage of YoY recruitment has changed over time. For example, if over the years settlement has spread to greater depths, beyond the current survey, the index could overstate predicted recruitment declines (Goode et al. 2019). Information on deep water settlement remains limited. While lobster postlarvae are responsive to thermoclines and tend to restrict habitat selection dives to depths with temperatures >12°C (Annis et al. 2013), and settlement has been reported as deep as 80 m (Wahle et al. 2013a), no long‐term data are available to evaluate changes in the depth distribution of settlement over time.

Broadly, all predictive models relying on life history information and ecological parameters are subject to sampling bias and process error, which can impair accurate predictions (Patterson 2001). Process error can arise from uncertainty in biological parameters (Newman et al. 2006); in our case natural mortality and growth function. We evaluated the model sensitivity to variability in growth using exploratory correlation (Appendix S1: Table S3).

Estimating age at size is one of the greatest challenges in developing forecasting tools for long‐lived crustaceans, such as lobster. We estimated age at recruitment to the fishery based on an understanding of growth variability. By virtue of a clear size mode in the population at the end of the settlement season, we could assign individuals less than a certain size to the 0‐yr class with near certainty (Incze and Wahle 1991). From this starting point, it is necessary to apply a logistic function to estimate the proportion recruiting to the fishery at age (Wahle et al. 2004). A novel aspect of our study was to scale the mean age at fishery recruitment parameter (*r*) of the logistic growth function to temperature based on empirical mark–recapture studies across the species’ thermal range (Appendix S1).

Estimating natural mortality also remains elusive. For our purposes, incorporating a base rate (ASMFC 2015), and adding shell disease as a proxy for elevated natural mortality where it became prevalent improved our model fit substantially, as previously shown by Wahle et al. (2009). This does not assume that all lobsters afflicted with shell disease die; we simply assert that fishery recruitment trends are proportional to disease prevalence, in addition to settlement (Wahle et al. 2009). We also do not rule out the possibility that elevated predation in the southern part of lobster's range may also play a role (Wahle et al. 2009, 2013b). In a changing climate, the northward advancement of potential predators, competitors, and pathogens, may change the landscape of natural mortality in the future.

One risk of developing forecasting models at relatively small sub‐stock scales is that exchange of individuals among areas or changes in fishing effort may confound settlement‐based prediction of landings trends. Post‐settlement movements along the bottom could subsidize fishery recruitment or expanded fishing effort beyond the immediate boundaries of the area could explain unpredicted landings trends, such as in northern Massachusetts where our forecasts underestimated recent landings. In these cases, aggregating study areas that encompass migratory and variable fishing effort dynamics may be warranted.

Recent long‐term projections under current climate change scenarios suggest that American lobster recruitment potential in the Gulf of Maine may be near its peak in the current decade, and will decline as temperatures rise above optimal limits (Le Bris et al. 2018). Notwithstanding the possibility that thermal habitat expansion could have the mitigating effect described above, our evidence of a recent widespread downturn in YoY settlement at a time of historic highs in spawning stock, suggest that recruitment success per egg may already be on the decline. The specific mechanism by which such widespread decline could occur is not understood, but changes in the pelagic environment and food web associated with warming could impact planktonic larval stages (Perretti 2017, Carloni et al. 2018).

How should stakeholders use this forecasting tool? We recommend that stock assessment community and fishery managers use this information in concert with other indicators of the health of the fishery, as an independent early warning system. A central aim of lobster fishery management is to maximize egg production though conservation of broodstock. Although healthy broodstock is no guarantee of high recruitment, high recruitment cannot happen in the absence of a healthy broodstock. Indeed, multidecade life history models suggest that the climate‐mediated lobster fishery collapse in southern New England might have been forestalled had more protections of the reproductive stock been in place (Le Bris et al. 2018).

Striving to develop and refine predictive tools in fisheries is worthwhile because of the lead time it gives stakeholders to consider their choices. Future declines in the American lobster may be especially acute and disruptive because of over‐reliance of segments of the coastal economy on this single fishery (Steneck 2011). However, all predictive models should be assessed critically and in the context of other demographic indicators. Incorporating such a forecasting approach into the suite of tools available may enhance the capacity of fisheries scientists, managers, and industry members to adapt to a changing environment.

## Data Availability

Data used to generate tables and graphs, as well as MatLab code files used for the model described in this paper, has been made publicly available through the University of Maine's Dataverse data management system: http://dataverse.acg.maine.edu/dvn/dv/Oppenheim

## Note

1
http://www.fishbase.org/search.php
