# Supplementary material for: The cresting wave: larval settlement and ocean temperatures predict change in the American lobster harvest
Source: Ecol Appl. 2019 Oct 21;29(8):e02006. doi: 10.1002/eap.2006 (PMC6916173; doi:10.1002/eap.2006)
Supplement: Supplementary file 1 [file EAP-29-na-s001.pdf]

SUPPORTING INFORMATION: APPENDIX S1

The cresting wave: larval settlement and ocean temperatures predict change in the American lobster harvest

Noah G. Oppenheim[1,2], Richard A. Wahle[1*], Damian C. Brady[1], Andrew Goode[1], Andrew Pershing[3]

1. University of Maine School of Marine Sciences, Darling Marine Center, Walpole, ME 04573, USA. Wahle Email: richard.wahle@maine.edu, Tel: 207 841 7723; Brady Email: damian.brady@maine.edu, Tel: 207 312 8752, Goode Email: andrew.goode@maine.edu
2. Institute for Fisheries Resources, 991 Marine Drive, San Francisco, CA 94129, USA. Email: noah@ifrfish.org, Tel: 415 561-3474
3. Gulf of Maine Research Institute, Commercial Street, Portland, ME 04101, USA. Email: apershing@gmri.org, Tel: 207 228 1656

* Corresponding Author

Journal: ***Ecological Applications***

Anticipated publication year: 2019

Data Sources

American Lobster Settlement Index: ALSI is a scuba-based benthic survey designed to assess the abundance of newly settled young-of-year (YoY) lobsters repopulating rocky coastal nurseries every year. Because the YoY are readily identified as a size mode in the population, and no direct aging methods are available, ALSI is the only monitoring program that measures year-class strength for this species (Wahle and Incze 1997, Incze et al. 1997). Initiated in 1989, the survey has expanded to approximately 100 annually monitored fixed sampling sites nested

within 26 larger study areas throughout the American lobster's range, from Rhode Island to Newfoundland (Wahle et al. 2013a; Fig. 1). Surveys are conducted at the end of the postlarval settlement season when YoY lobster have finished recruiting to nurseries (late August in the southwest to late October in the northeast). At each sampling site, divers use airlift suction samplers in cobble habitat to make collections from between 12 and 20 0.5 m^2 quadrats to capture juvenile lobsters over a range of sizes and ages. Population densities are standardized to numbers per square meter. Densities are averaged among sites to give a study area mean YoY density ± 1 standard error (Fig. S1).

Here we evaluate the predictive power of the ALSI time series from ten study areas in the Gulf of Maine, Bay of Fundy and southern New England representing the longest time series of the survey up through the 2015 survey. Growth rates are naturally variable and temperature dependent (Aiken and Waddy 1986) resulting in lags of ~5-10 years between settlement and recruitment to the fishery. We therefore only included study areas with time series of sufficient length to generate at least seven years of recruitment to the fishery comprising the full complement of contributing age classes (Table S1).

Landings: Lobster landings were used in this study as a proxy for fishery recruitment and served as an independent estimate of recruitment against which we could validate trends in the predicted recruitment index. Landings time series were obtained from state (Rhode Island Department of Environmental Management, Massachusetts Department of Marine Fisheries, and Maine Department of Marine Resources) and Canadian Federal (Department of Fisheries and Oceans) marine resource management agencies (Table S1; Fig. S2). We used landings time series from fishery statistical reporting areas most closely corresponding to ALSI study areas.

We justify the use of landings as our proxy for abundance for three reasons: First, the American lobster fishery is generally considered to be a recruitment-limited fishery, with approximately 85% of lobsters landed in any given year being new recruits to the fishery (ASMFC 2015). Fishing effort throughout New England represents an unparalleled collective sampling by thousands of fishermen. Second, fishery independent trawl surveys have not been conducted uniformly or for long enough over the region of interest to provide adequate model validation; nor was there sufficient survey coverage at the relatively small scale of our study areas to be representative of area-specific trends. Finally, expressing forecasts as landings facilitates translating our output to industry stakeholders and policy makers. While landings can be sensitive to economic, environmental and biological forces that influence fishing effort, on the whole, they have correlated strongly with fishery-independent surveys in the region (Boudreau et al. 2015).

Bottom Temperature: Because lobster growth and development are strongly temperature dependent (Makenzie 1988, Drinkwater *et al.* 1996, Wahle and Fogarty 2006) we included temperature as a variable in the predictive models. We used the third generation of the Northeast Coastal Ocean Forecast System (NECOFS) Finite Volume Community Ocean Model for the Gulf of Maine (FVCOM-GOM) 30 year hindcast model to generate annual average bottom temperature time series for each of the ALSI study areas. FVCOM-GOM is the circulation model component of NECOFS and consists of an unstructured grid, data-assimilative ocean circulation model for the Gulf of Maine and southern New England study areas (He *et al.* 2005; Chen *et al.* 2006). FVCOM-GOM has particularly high resolution in the nearshore environment (~20 m – 1 km) and includes a long term (1979-2014) hindcast of bottom water temperature. Recent validation studies of the hindcast have shown it to be a reliable estimate of bottom water

temperatures at the temporal (multi-year) and spatial (fishery statistical reporting area) scales of this analysis (Manning 2012; Li et al. 2017). All FVCOM-GOM nodes within a ~10 km grid corresponding to coastal shelf waters adjacent to ALSI study sites were averaged for hourly bottom temperatures. We then aggregated the hourly bottom temperatures within each study area to give an annual mean (Fig. 2a).

Natural Mortality and Shell Disease: There are no reliable empirical estimates of natural mortality for the American lobster, much less at the spatial and temporal resolution of this study. We therefore used the conventional value of natural mortality used in stock assessment (ASMFC 2015), a baseline instantaneous mortality of 0.15 yr^{-1} for all cohorts and study areas. Shell disease prevalence data from at-sea surveys of commercial catch conducted by state marine resource management agencies were used as a proxy for changes in disease-based mortality (e.g., Glenn and Pugh 2006; Fig. 2b). Shell disease has not been detected in Canadian surveys to date. The proportion of lobsters less than the legal size (83 mm CL) showing any sign of shell disease was added to the base mortality to provide an augmented annual mortality rate.

Process Model

All modeling and statistical analysis were conducted in MatLab v.9.0. The fishery recruitment index R for a given year t is equal to the sum of the fractional contribution of cohorts at age a to fishery recruitment in year t, such that:

$$R_t = \sum_a P_a \cdot n_{t-a} \cdot (1 - m_t)^a \qquad \text{(Equation S1)}$$

where n_{t-a} is YoY settlement density in year t-a, and m_t is natural mortality in year t. We held natural mortality constant at an annual proportion of 0.139 yr^{-1}, equivalent to an instantaneous rate of 0.15 yr^{-1}, for all study areas, except where shell disease incidence rates added to baseline mortality to give the year-specific m_t term. A constant age-specific mortality is assumed in this

case for lack of empirical estimates under the assumption that high shelter fidelity in early stages offsets the higher risk of predation (Wahle et al. 2013b).

We approximated the proportion *P* of a cohort entering the fishery at age *a* with a modified version of a logistic function described by Wahle et al. (2004):

$$P_a = \frac{1}{1 + e^{-b(-r_{t,i}+a)}} \qquad \text{(Equation S2)}$$

where *r* is the estimated mean age at fishery recruitment and *b* is the coefficient determining the slope of the logistic curve. An important innovation was to make *r* temperature dependent. We derived this term from probabilistic stepwise growth models specified for three thermally contrasting study areas along the thermal gradient of interest: southern New England, Midcoast Maine, and Beaver Harbour, New Brunswick (Bergeron 2011, based on Chen et al. 2005). The models were parameterized using data from mark-recapture studies conducted in the three areas. To estimate *r* and its uncertainty for each area, we conducted 1000 Monte Carlo simulations using observed sex- and size-specific variability in molt frequency and increment for each of the three areas (Table S2). In the simulation, lobsters recruited to the fishery when they first reached or exceeded harvestable size (83 mm carapace length). To convert *r* from a static to a temperature-dependent variable, we evaluated the relationship between the area-specific *r* and the corresponding mean bottom temperature for the same area (Fig. 2c). We used the slope and intercept from the resulting regression ($r^2 = 0.52$; $p < 0.0001$; RMSE = 1.48) in the following equation to estimate mean fishery recruitment age at temperature:

$$r_{t,i} = 0.98(T_{t,i}) + 15.8 \qquad \text{(Equation S3)}$$

where *T* is bottom water temperature for each year *t* and study area *i*.

To set the slope of the logistic function (*b),* we conducted exploratory correlations to optimize the parameter for each study area (Fig. 2d). The domain of the *b* parameter was constrained to eliminate unrealistically early recruitment to the fishery, such as at ages 1 or 2 years. The model was relatively insensitive to changes in *b,* which determines the slope of the logistic curve, relative to changes in the mean age of recruitment (Table S3). This is demonstrated by the broad domain of *b* terms over which statistically significant relationships between forecasts and landings were found.

Monte Carlo Simulations: For study areas with statistically significant hindcast models, we used a Monte Carlo simulation procedure to represent the uncertainty in predicted recruitment (Smith and Addison 2003). This approach allowed us to incorporate parameter error estimates into model outputs. We estimated normally distributed statistical error for two key parameters in the recruitment model, YoY density (n) and average age-at-fishery recruitment (r), that originated from empirical survey data. Error for cohort density was generated from the among-site variability in the settlement index each year. Error for age-at-fishery recruitment was estimated from the empirical tagging data that informed the area-specific logistic function giving the proportion recruiting to the fishery at age (Bergeron 2011). This procedure was repeated 1000 times for each study area over a biologically plausible range of values of the logistic slope parameter *b.* Model outputs provided probability distributions of predicted recruitment to the fishery for each study area.

Table S1. Study area descriptive data.

Study Area	Year of initiation	Number of sites	YoY size threshold (mm CL)	Correspondent statistical reporting area	Reporting/collaborating agency
Beaver Harbour, NB	1991	4	13	Canada LFA 36	DFO (Canada)
Jonesport, ME	2001	4	13	ME Zone A	ME DMR
Mount Desert, ME	2000	5	10.5 '95-'98; 13 ≥ '99	ME Zone B	ME DMR
Pen Bay East, ME	2000	5	13	ME Zone C	ME DMR
Pen Bay West, ME	2000	6	13	ME Zone D	ME DMR
Midcoast, ME	1989	10	10.5	ME Zone E	ME DMR
Casco Bay, ME	2000	5	10.5	ME Zone F	ME DMR
York ME	2000	5	10.5	ME Zone G	ME DMR
Northern Massachusetts	1995	6	12	Mass. Area 3	MA DMF
Southern New England	1992	12	13	Mass. Area 14	MA DMF & RI DFW

Table S2. Stepwise growth model-based estimates of median age at recruitment to the fishery (≥83 mm CL) and quantiles by sex for the three oceanographically contrasting study areas with empirical growth data. The recruitment model (equation 2.1) used the average of the male and female values.

Model	Sex	N	10% Quantile	25% Quantile	Median	75% Quantile	90% Quantile
Beaver Hbr, NB	Male	1000	7.68	8.59	9.58	10.73	11.96
	Female	1000	6.45	7.19	8.18	9.08	10.07
Midcoast ME	Male	1000	5.55	6.37	7.36	8.40	9.25
	Female	999	5.38	6.12	7.03	8.01	8.84
SNE	Male	996	3.64	4.13	4.79	5.53	6.27
	Female	1000	3.80	4.46	5.20	6.27	7.08

Table S3. Model selection and sensitivity to the slope (parameter *b*) of the logistic function (Equation 2.2). Values of *b* were set to a biologically plausible range, and models were created for incremental values of *b* within the range. The best models were selected based on maximum correlation between recruitment forecasts and landings. SD = Shell disease.

Study Area	Best model	Domain of Significant *b*	Optimal *b*	r	p-value
Beaver Hbr, NB	Fixed temp	0.41 - 2.46	0.76	0.97	<0.0001
Jonesport, ME	Variable temp	0.41 - 2.46	0.46	0.92	0.0010
MDI, ME	Fixed temp	0.41 - 2.46	0.56	0.97	<0.0001
Pen Bay E, ME	Fixed temp	0.41 - 2.46	0.41	0.95	<0.0001
Pen Bay W, ME	Fixed temp	0.41 - 0.76	0.41	0.80	0.0087
Midcoast, ME	Fixed temp	0.41 - 1.11	0.41	0.75	0.0002
Casco Bay, ME	Fixed temp	0.41 - 2.46	1.36	0.72	0.0217
York, ME	Fixed temp	-	0.41	-0.45	0.2169
N. MA.	Variable temp	0.41 - 2.46	0.91	0.75	0.0008
S. New England	Variable temp + SD	0.41 - 2.46	2.26	0.71	0.0076

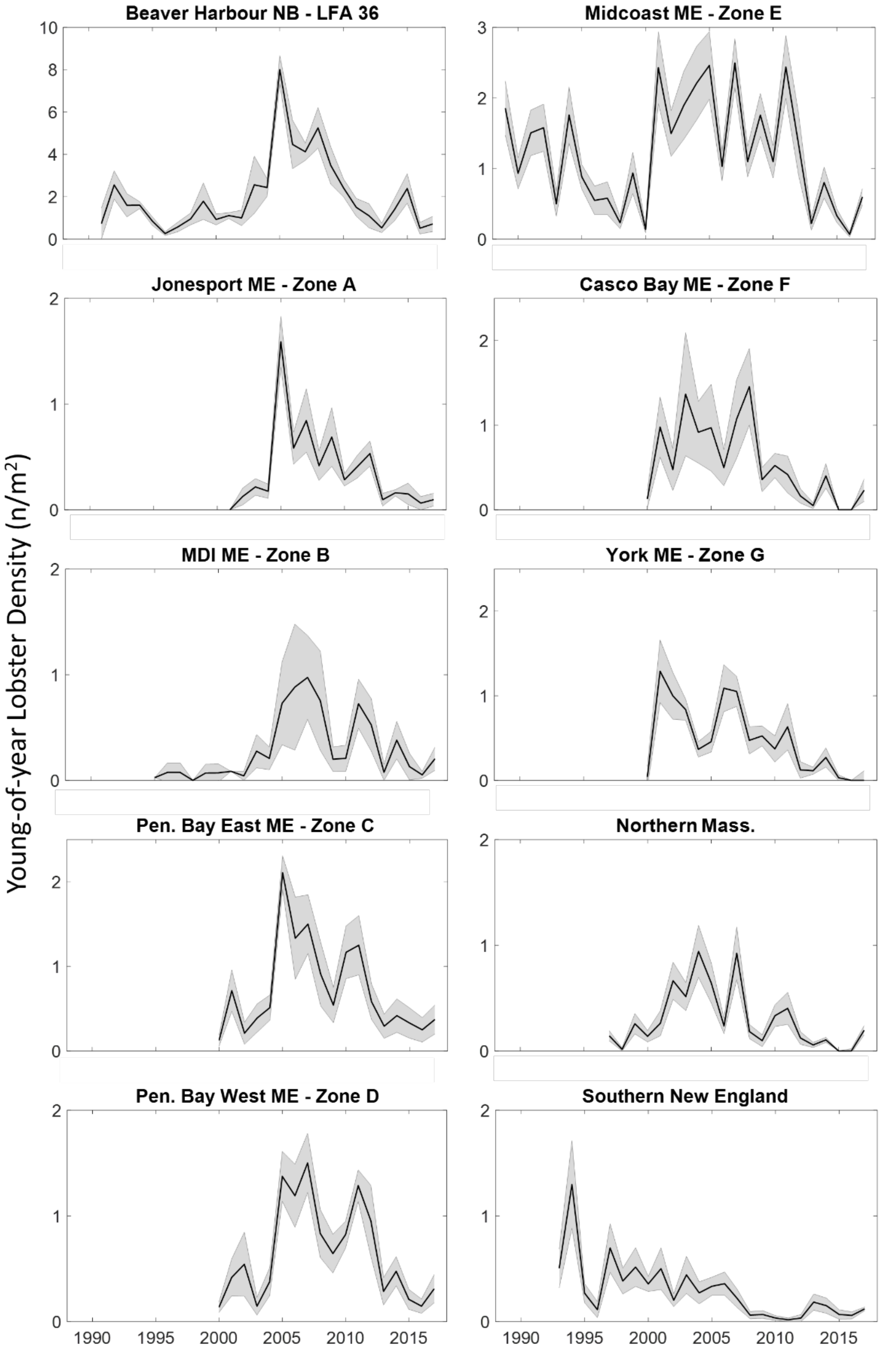

Figure S1. American Lobster Settlement Index settlement time series ± 1 standard error for the 10 study areas through 2017.

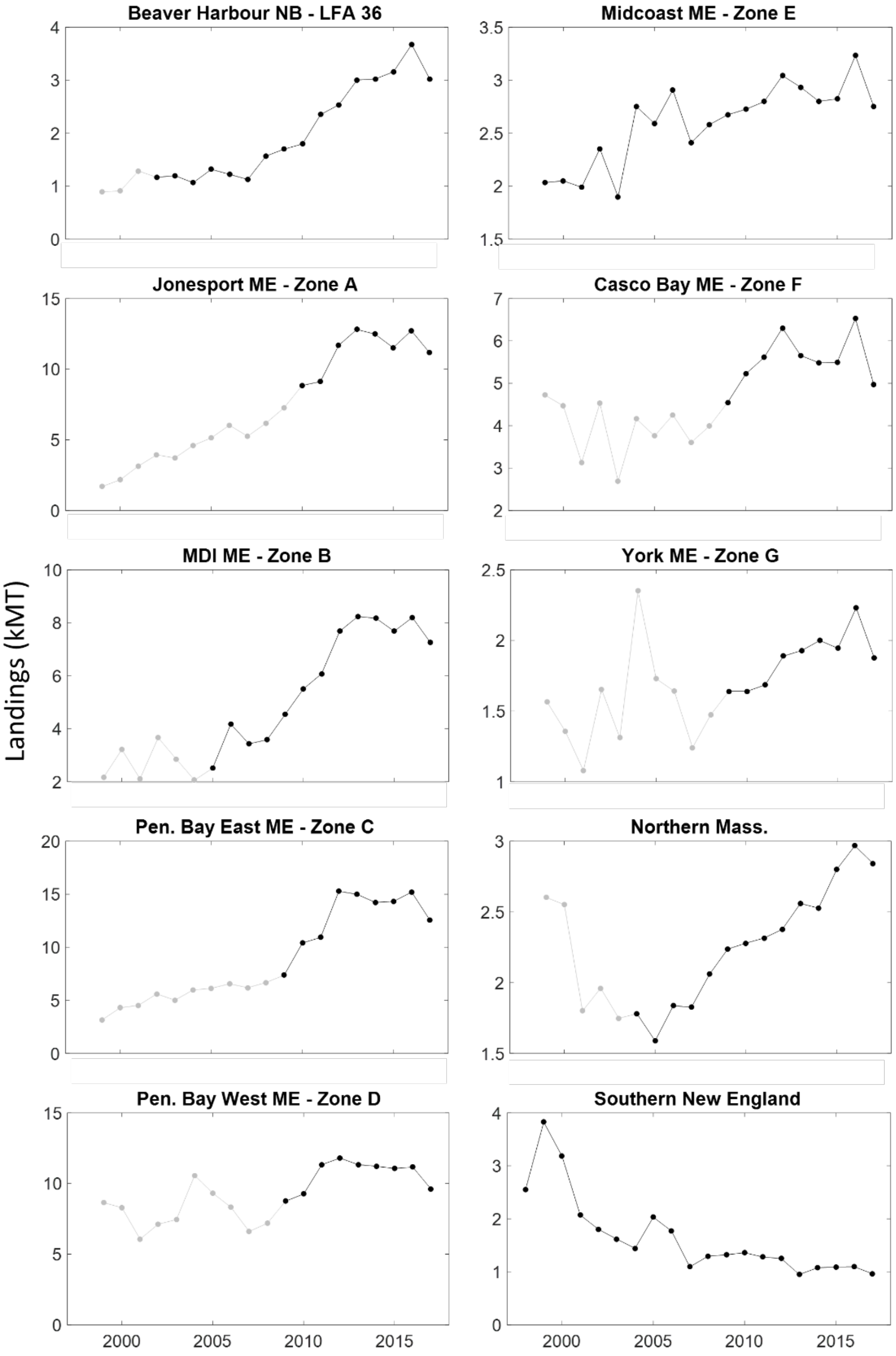

Figure S2. Lobster landings time series through 2017. Data are from fishery statistical reporting areas corresponding to ALSI study areas. Years within the domain of the longest hindcast are shown in black.

Literature Cited for Appendix S1

Aiken, D. E., and S.L. Waddy. 1986. Environmental influence on recruitment of the American lobster *Homarus americanus*: a perspective. *Canadian Journal of Fisheries and Aquatic Sciences 43*: 2258-2270.

Atlantic States Marine Fisheries Commission (ASMFC). 2015. American lobster stock assessment and peer review report. Retrieved from https://asmfc/egnyte.com/dl/kJfBicPSNR

Bergeron, C. E. 2011. Research on lobster age-size relationships: Developing regionally specified growth models from meta-analysis of existing data. Master's thesis, UMaine.

Boudreau, S. A., S.C. Anderson, and B. Worm 2015. Top-down and bottom-up forces interact at thermal range extremes on American lobster. *Journal of Animal Ecology 84*: 840-850.

Chen, Y., M. Kanaiwa and C. Wilson. 2005. Developing and evaluating a size-structured stock assessment model for the American lobster, *Homarus americanus*, fishery. *New Zealand Journal of Marine and Freshwater Research 39*: 645-660.

Glenn, R. P., and T. L. Pugh. 2006. Epizootic Shell Disease in American Lobster (*Homarus americanus*) in Massachusetts Coastal Waters: Interactions of temperature, maturity, and intermolt duration. *Journal of Crustacean Biology* 26:639-645.

He, R., D. J. McGillicuddy, D.R. Lynch, K.W. Smith, C.A. Stock, and J.P. Manning. 2005. Data assimilative hindcast of the Gulf of Maine coastal circulation. *Journal of Geophysical Research: Oceans (1978–2012)*, *110*(C10).

Incze, L. S., R.A. Wahle, and J.S. Cobb. 1997. Quantitative relationships between postlarval production and benthic recruitment in lobsters, *Homarus americanus*. *Marine and Freshwater Research 48*: 729-744.

Li, B., K.R. Tanaka, Y. Chen, D.C. Brady, and A.C. Thomas. 2017. Assessing the quality of bottom water temperatures from the Finite-Volume Community Ocean Model (FVCOM) in the Northwest Atlantic Shelf region. *Journal of Marine Systems 173*(Supplement C): 21-30. doi:https://doi.org/10.1016/j.jmarsys.2017.04.001

Manning, J. 2012. Environmental monitors on lobster traps Phase VII: Validating ocean models (Final Report). Retrieved from http://www.nefsc.noaa.gov/epd/ocean/MainPage/lob/emolt_phaseVII_final.pdf

Wahle, R. A., and L.S. Incze. 1997. Pre-and post-settlement processes in recruitment of the American lobster. *Journal of Experimental Marine Biology and Ecology 217*: 179-207.

Wahle, R.A., C. Bergeron, J. Tremblay, C. Wilson, V. Burdett-Coutts, M. Comeau, R. Rochette, P. Lawton, R. Glenn, and M. Gibson. 2013a. The geography and bathymetry of American lobster benthic recruitment as measured by diver-based suction sampling and passive collectors. *Marine Biology Research* 9: 42-58.
